# High throughput phenotyping dataset related to seed and seedling traits of sugar beet genotypes

**DOI:** 10.1016/j.dib.2020.105201

**Published:** 2020-01-27

**Authors:** Sylvie Ducournau, Aurélie Charrier, Didier Demilly, Marie-Hélène Wagner, Ghassen Trigui, Audrey Dupont, Sherif Hamdy, Karima Boudehri-Giresse, Laurence Le Corre, Laurence Landais, Angélique Delanoue, Dorothée Charruaud, Karine Henry, Nicolas Henry, Lydie Ledroit, Carolyne Dürr

**Affiliations:** a Groupe d’Etude et de contrôle des Variétés Et des Semences (GEVES), 25 rue Georges Morel, 49071, Beaucouzé, France; b INRAE, UMR URGI Unité de Recherche Génomique Info, Route de Saint-Cyr, 78000, Versailles, France; c Ets Florimond Desprez, BP 41, 59242, Cappelle-en-Pevele, France; d INRAE, UMR IRHS Institut de Recherche en Horticulture et Semences, 42 rue George Morel, 49071, Beaucouzé, France

**Keywords:** Seed, Seedling, Germination, Heterotrophic growth, Automated phenotyping, Sugar beet, Genotypes

## Abstract

Several seed and seedling traits are measured to evaluate germination and emergence potential in relation with environmental conditions. More generally, these traits are also measured in the field of ecology as simple traits that can be correlated to other adaptative traits more difficult to measure on adult plants, as for example traits of the rooting system. Methods were developed for deep high throughput phenotyping of hundreds of genotypes from dry seed to the end of heterotrophic growth. The present dataset comes from a project on genotyping and phenotyping of populations of genotypes, with different geographic and genetic origins so as to increase genotypic diversity of sugar beet in terms of germination and early growth traits, evaluated at low temperatures. Data were collected in relation to the creation of the first sugar beet crop ontology. This dataset corresponds to the first automated phenotyping of a population of 198 genotypes and 4 commercial control varieties and is hosted on INRAE public depository under the reference number doi.org/10.15,454/AKNF4Q. The equipment and methods presented here are available on a phenotyping platform opened to collaborative research and adaptable for specific services for characterizing thousands of genotypes on different crops or other species. The phenotyping values can also be linked to genomic information to study the genetic determinism of the trait values.

**Table:** Specifications Table

Subject	Agricultural and Biological Sciences (General)
Specific subject area	Sugar beet seed morphology, germination and heterotrophic growth
Type of data	Table
How data were acquired	Data were acquired on the phenotyping platform PHENOTIC https://www6.inra.fr/phenotic/). The laboratory equipment on this platform includes: - a tomograph NSI X-50 (North Star Imaging, Minnesota, USA) - automated tools measuring germination (Multicam, ESEO, Angers, France) - automated equipment measuring heterotrophic seedling growth (Eloncam, ESEO, Angers, France) - 3D image processing: using Avizo image processing modules where the scripts were written in TCL scripting language (Ousterhout, TCL/TK) and MATLAB (The MathWorks, Inc., Natick, Massachusetts, United States). - 2D image processing of seed germination: using Fiji image processing package based on ImageJ, http://fiji.sc/.
Data format	Raw
Parameters for data collection	3D X-ray data were acquired on dry seeds. All the measurements for seed germination and seedling heterotrophic growth were made at low temperatures, which is a main limiting condition for sugar beet at these early stages.
Description of data collection	Laboratory experiment on paper layers, non limiting water, low temperatures, automated image acquisition for measurements on seeds and seedlings.
Data source location	The genotype population was obtained from a cross between an elite commercial genotype used in European sugar beet (*Beta vulgaris* L.) grown area and an exotic accession of *Beta vulgaris maritima* from Denmark. Institution: Florimond Desprez; City/Town/Region: Cappelle-en Pévèle; Country: France. Latitude and longitude for collected samples 50.5167; 3.1667
Data accessibility	Repository name: URGI Plant and Fungi Dataverse Data identification number: https://doi.org/10.15454/AKNF4Q Direct URL to data: https://doi.org/10.15454/AKNF4Q

**Table:** 

**Value of the Data** • Seed and seedling traits are increasingly measured in the field of ecology as simple traits that can be used to describe species diversity. A deeper phenotyping of genetic diversity in crops is also necessary to better understand their tolerance to varied environmental conditions and determine more precise breeding traits. Measurements on seed or seedling traits are easier than on an adult plant. They can be correlated to more general adaptative traits to environmental conditions [1,2]. This is the case, for instance, for the temperature or water potential responses [3] or traits of the young root system [4,5]. • Several methods were developed for the deep high throughput phenotyping of hundreds of genotypes from dry seed to the end of heterotrophic growth [6]. This is a first example of automated measurements from dry seed to young seedling carried out on a progeny (elite X exotic) of 198 sugar beet genotypes (plus 4 standards as references). Such set of data can be reproduced for other species in order to characterize seed and seedlings in their early developmental stages. • The methods presented here can now be routinely used for the phenotyping of several thousands of genotypes and other species on a platform accessible to users [7]. • The dataset was collected in relation to the sugar beet crop ontology which was also created within the same project [8]. The data set provides the range of values measured for each variable on a large population of genotypes. The ontology defines a general international framework to collect and compare variables' values. The range of values measured here could be compared with measurements on other genotypes at intra- and inter-species level to determine the range of variation of these traits' values and provide a better understanding of adaptation of genotypes or species to different agro- or ecosystems [9,10].

## 1 Data description

The dataset contains 28 variables measured for each genotype. Data is represented by three groups of variables (Table 1) measured on a population of 198 sugar beet genotypes plus 4 standard commercial varieties. This data was obtained at high throughput on automated equipment. The first group of variables describes the dry seed. An innovative tool (X-ray microtomography) was used to investigate the internal anatomy of sugar beet seeds. This technology generates high-resolution images of internal seed structures. An automated image processing protocol was developed to extract data on traits of interest. The obtained data provide precise measurements of volume, surface area and shape of seed embryo, perisperm and seed coat. The second group describes seed germination speed measured as mean germination time and time to reach 50% and 70% germination, and final germination rate at three temperatures 5 °C, 10 °C and 20 °C, obtained by automated acquisition and analyzes of images. The third group describes individual seedling elongation rate measured 7 days after seed germination at 10 °C by automated image acquisition under inactinic lights. These variables are consistent with the sugar beet international crop ontology.

**Table 1:** Measured variables on sugar beet genotypes with the PHENOTIC platform equipments.

Phenotyping device	Variable identification code in the ontology	Variable name	Variable	Unit
Tomograph	CO_333:1000327	SeedMass	Seed Mass	mg
	CO_333:1000324	PerispVol	Perispersm Volume	mm^3^
	CO_333:1000323	PerispSurfArea	Perispersm Surface Area	mm^2^
	CO_333:1000389	PerispShapeVA3d	Perispersm Shape 3d	No unit
	CO_333:1000390	PerispSpDiameter	Perispersm Spherical Diameter	mm
	CO_333:1000320	EmbVol	Embryo Volume	mm^3^
	CO_333:1000319	EmbSurfArea	Embryo Surface Area	mm^2^
	CO_333:1000391	EmbShapeVA3d	Embryo Shape 3d	No unit
	CO_333:1000392	EmbSpDiameter	Embryo Sperical Diameter	mm
	CO_333:1000325	SeedCoatVol	Seed Coat Volume	mm^3^
	CO_333:1000317	CoatSurfArea	Seed Coat Surface Area	mm^2^
	CO_333:1000393	SeedCoatShapeVA3d	Seed coat Shape 3d	No unit
	CO_333:1000394	SeedCoatSpDiameter	Seed coat Spherical Diameter	mm
Multicam for Germination	**Germination at 5 °C**			
	CO_333:1000311	MGT 5 °C	Mean Germination Time	h
	CO_333:1000330	T50%	Time To Reach 50% Of Germination	h
	CO_333:1000330	T70%	Time To Reach 70% Of Germination	h
	CO_333:1000388	EGermRt 17 days at 5 °C	Early germination rate	h
	CO_333:1000321	GermFnlRt 28 days at 5 °C	Germination Final Rate	%
	**Germination at 10 °C**			
	CO_333:1000311	MGT 10 °C	Mean Germination Time	h
	CO_333:1000330	T50% 10 °C	Time To Reach 50% Of Germination	h
	CO_333:1000330	T70% 10 °C	Time To Reach 70% Of Germination	h
	CO_333:1000321	GermFnlRt 15 days 10 °C	Germination Final Rate	%
	**Germination at 20 °C**			
	CO_333:1000311	MGT 20 °C	Mean Germination Time	h
	CO_333:1000330	T50% 20 °C	Time To Reach 50% Of Germination	h
	CO_333:1000330	T70% 20 °C	Time To Reach 70% Of Germination	h
	CO_333:1000321	GermFnlRt 6 days 20 °C	Germination Final Rate	%
ElonCam	CO_333:1000386	HGerm 10 °C	Hour of germination at 10 °C	h
	CO_333:1000387	RadLg 10 °C	Radicle length 7 days after germination at 10 °C	mm

## 2 Experimental design, materials, and methods

An exotic accession of *Beta vulgaris maritima* from Denmark was crossed with a sugar beet elite pollinator (*Beta vulgaris* L.) from the Florimond Desprez company. Two successive backcrosses with another elite pollinator, from the same seed company, were completed, leading to 198 individuals that constituted the (elite X exotic) progeny. This population was completed by four commercial cultivars considered as controls and thus a total of 202 genotypes were phenotyped. A set of 28 variables were measured on the seeds of the 202 genotypes: 3D internal morphology of seeds, germination at several temperatures and automated acquisition of heterotophic seedling growth traits. These measurements were performed on a phenotyping platform PHENOTIC dedicated to instrumentation and imagery for seeds, seedlings and plants. The platform's activities are dedicated to both researchers and professionals, for specific services and collaborative research projects. Table 1 presents the list of the measured variables.

### 2.1 3D dry seed measurements

Tomography was performed on 25 seeds per genotype. Images were obtained using an NSI X-50 manufactured by North Star Imaging (Minesotta, USA). The system has a focus tube with a focal spot up to 1 μm, a flat-panel detector with a resolution of 256 x256 and an adjustable turntable. All seed samples were scanned at a voltage of 45 kV and a current of 178 μA, a rotation step of 0.5° acquiring 1080 images of the transmitted signal after passing through the object.

The reconstructed data are represented by 2D images combined into one image stack and ordered by their position in the z-axis of the imaged sample. The 3D volume reconstruction is obtained from multiple cross-section 2D images of the object, using the software supplied with the machine. The stack comprises (976 × 976 x 976) voxels in the (x, y, z) referential. The 3D image can either be visually interpreted or analyzed using image processing tools in order to extract relevant information.

An automated image processing pipeline (IPP) was developed to extract sugar beet seed features from raw data. This pipeline was developed using Avizo image processing modules where the scripts were written in TCL scripting language (Ousterhout, TCL/TK) and MATLAB (The MathWorks, Inc., Natick, Massachusetts, United States). The IPP consists of two successive tasks of morphological operations. The first task was designed to individualize the sugar beet seeds in each image ***I****xyz* into separate objects where each scan has a fixed number of 25 seeds. Some pre-processing steps such as noise removal and segmentation were applied to separate the voxels that correspond to sugar beet seeds from the sample holders, and also to generate 3D binary masks ***M****xyz* that represent only the voxels that belong to the sugar beet seeds according to equation (1). The resulting set of masks was obtained after the segmentation with a threshold τ which was experimentally fixed.

The original image ***I****xyz* was then masked using the generated set of binary masks ***M****xyz,* as shown in equation (2).

(1) $$M_{xyz}=\{\begin{array}{c} 0\;if\;I_{xyz}\leq \tau \\ 1\;if\;I_{xyz}>\tau \end{array}$$

(2) $$f\left(I_{xyz},M_{xyz}\right)=\left\{i_{1,xyz},i_{2,xyz}\ldots i_{25,xyz}\right\}$$

**Fig. 1:**
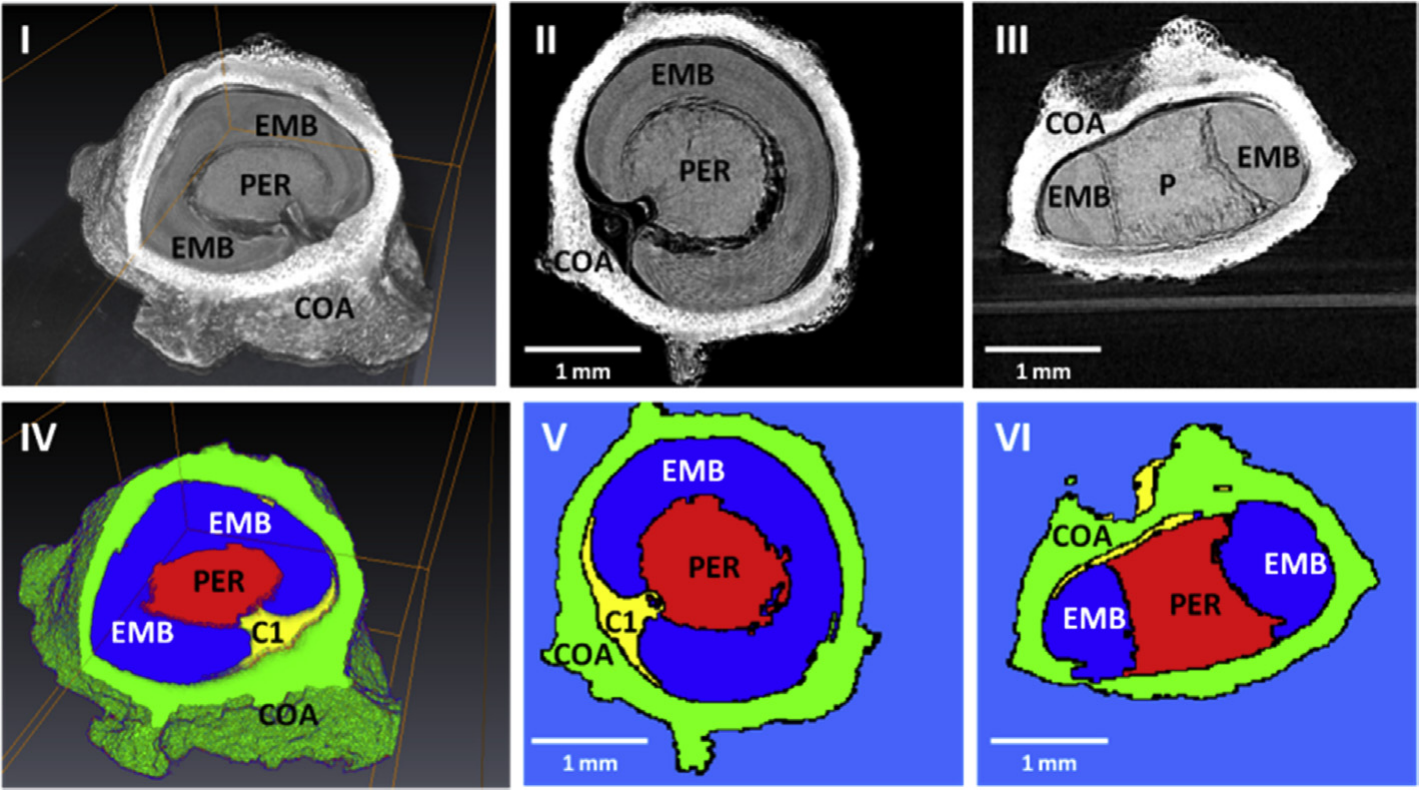
Raw data and image segmentation results of a beet seed. (I,IV) 3D volume rendering showing the structural components of the seed namely EMB for embryo, PER for perisperm, COA for seed coat, and C1 for internal empty space and with respectively transverse (II, V) and longitudinal (III, VI) cross-section views. (bar = 1mm).

The second task in the IPP aims to measure and estimate the traits and characteristics of the principal structural components (embryo, perisperm and seed coat) of sugar beet seeds. The mentioned regions of interest (ROI) of the seed's structure were segmented based on the differences in intensities induced by X-ray attenuation (Fig. 1). The marker-controlled watershed segmentation was then performed to separate seed's structure [11]. The markers used as inputs of the watershed operation were determined using the succession of morphological erosion and dilation of the binary image. Once separated, several features for each individual structure were measured.

The image processing workflow comprises also a filtering step to eliminate morphologically distorted measurements corresponding to dead, malformed or empty seeds. Multiple analyzis and features extraction of the embryo, perisperm and seed coat were carried out on the scanned images like estimating the volume, surface area, shape factor, filling factor and the spherical diameter. A total number of 14 variables were extracted for each component of the seed. The outputs of the procedure give directly a statistical summary (mean and standard deviation for 25 seeds) of each of the features.

The last version of the pipeline is a fully automated process that was intensively optimized and improved compared to the previous versions to reach 32 minutes to perform the IPP with an estimated phenotyping time of approximately 2.36 minutes per seed.

### 2.2 Germination at several temperatures

Germination time courses were recorded at 5, 10 and 20 °C using PHENOTIC automated germination tools [12]. Two pieces of equipment were used to phenotype seed germination at 5 °C, with a maximum of 1600 seeds each. Two other automated tools were used for 10 and 20 °C seed germination phenotyping. The temperature accuracy at the seedbed level was ±0.5 °C. Two replicates of 25 seeds were randomly sown on top of blotter (GE Heathcare 3644) continuously supplied with demineralised water in a Jacobsen tank system. Seeds were germinated in the dark except when images were taken every 2 hours (at 10 and 20 °C) and every 4 hours (at 5 °C) with less than 1 min of light. Seed germination was detected automatically by image analyzis. The individual seed germination time was determined by seed movement and radicle protrusion. The mean germination time [13] was calculated for the data in each experiment as well as percentile germination parameters (time to reach 50% and 70% of germination, see Table 1) that were extracted from germination curves. Early counts after 17 days at 5 °C was measured as an additional evaluation of seed vigor. Final germination rates were also reported after 28, 15 and 6 days, respectively, at 5 °C, 10 °C and 20 °C.

### 2.3 Heterotrophic seedling growth, radicle elongation

A growth chamber was specifically equipped for measurements of seedling heterotrophic growth. A method commonly used for mimicking darkness for plants is to observe them under green light, as the plant photoreceptors are assumed to be not activated under wave-lengths in the 515–550 nm range and are sensitive only either to red/far red or blue wavelengths expositions respectively [14,15]. Green LEDs, with narrow light spectrum in the range 515–550 nm were used to light the seeds or seedlings during image acquisition. A color camera (Gigabit Ethernet camera Prosilica GC2450C, 5.0 Megapixel camera with Sony ICX625 CCD sensor, 2448 × 2050 pixels) was used with a Fujinon C-Mount 5MP 2/3″ 16mm f/1.6 lens, allowing a resolution of 9pixels/mm at seed and seedlings locations. The image acquisition by this camera generally lasts 0.1 second and is synchronized with an on/off switch of light. Images are taken at intervals chosen by the operator, starting at a given time after sowing, with a limit of at least 1 h between two images. Interval between images were chosen according to the growth temperature and to limit the number of light flashes received by seedlings. Two rails with 20 plastic boxes (15 × 15 cm) loaded on each one with precisely determined positions, were available to grow seedlings. The back of the boxes was covered with a blue paper saturated with demineralised water. In each box, ten seeds were sown and covered with a second sheet of blue paper saturated with water, for seed imbibition and also mechanical support to prevent seedlings from falling during their growth. Thus, seedlings were grown almost vertically (with an inclination of 10°) and follow their natural gravitropism during their growth. A plate (30 × 30 cm) equipped with LED green lights and the camera were arranged on a backlight mode, with the box placed between the camera and the LEDs. LEDs were switched on in synchronization with image capture. Two cameras and plates of LEDs (one for each rail with plastic boxes) moved together and precisely stopped at the 20 box positions to take images of these boxes. The camera positioning was very precisely controlled, with one pixel precision, by the software piloting image acquisition. The entire system was placed in a growth chamber, with controlled temperature (range 10–30 °C). Green channels of images were saved and analyzed at the end of experiment with ImageJ software. The time for germination of each seed was determined as the hour at which a radicle becomes visible on the images. Starting time from each individual seed germination, individual seedling length of each organ (shoot and radicle) can then be measured and elongation curves can be drawn for each seedling, starting from its own germination time. The different seedlings parts were separated by visual inspection based on changes in grey levels and/or angles between organs. The possibility to look at several successive images also helps in separating plant parts. For the experiment described here, temperature was 10 °C. Images were taken every 4 h for 21 days, leading to a total of 20,160 images. The germination time of each seed was determined (Hgerm) and measurement of the seedling radicle length (RadLg) was performed exactly seven days (148 hours) after individual seed germination time.
